# Supplementary material for: Emergence and control of photonic band structure in stacked OLED microcavities
Source: Nat Commun. 2021 Oct 20;12:6111. doi: 10.1038/s41467-021-26440-3 (PMC8528838; doi:10.1038/s41467-021-26440-3)
Supplement: Supplementary file 4 — Supplementary Data 1 [file 41467_2021_26440_MOESM4_ESM.zip › OLED Simulation v2-1/OLED Simulation/Materials Data/Materials Database/rii-database-2019-02-11.zip!/database/info/main/CeF3.html]

# Cerium trifluoride, CeF_3_

## Other names

- Cerium(III) fluoride
- Cerium fluoride

## Mineral

- Fluocerite-(Ce)

## External links

- Cerium trifluoride - NIST Chemistry WebBook
- Cerium(III) fluoride - Wikipedia
- Cerium trifluoride - WebElements
- Fluocerite - Wikipedia
